# Early-onset digestive system cancers attributable to the GBD composite dietary risk in China, 1990–2023: burden, trends, and projections

**DOI:** 10.3389/fmed.2026.1972611

**Published:** 2026-10-07

**Authors:** Ping Li, Xiao Tong

**Affiliations:** 1 Department of Anorectal Traditional Chinese Medicine, West China Hospital Sichuan University Jintang Hospital, Jintang First People’s Hospital, Chengdu, Sichuan Province, China; 2 Department of Traditional Chinese Medicine Rehabilitation, Jintang Second People’s Hospital, Chengdu, Sichuan Province, China

**Keywords:** age-period-cohort analysis, dietary risks, digestive system neoplasms, disease burden, early-onset cancer

## Abstract

**Background:**

Digestive system cancers attributable to the aggregate dietary risk category defined by the Global Burden of Disease (GBD) study represent an important public health concern among Chinese adults aged 25–49 years. This study evaluated their burden, temporal trends, age-period-cohort patterns, and model-based projections.

**Methods:**

Data on deaths and disability-adjusted life years (DALYs) attributable to the GBD aggregate dietary risk category were obtained for Chinese individuals aged 25–49 years from 1990 to 2023. Joinpoint regression was used to analyze temporal trends, an age-period-cohort (APC) model was used to estimate age, period, and cohort effects, and a Bayesian age-period-cohort (BAPC) model was used to project the burden through 2050.

**Results:**

In 2023, these cancers were associated with 7,134 deaths and 349,764 DALYs. The burden among males was more than twice that among females. Age-standardized mortality and DALY rates declined significantly from 1990 to 2023 across all analyzed age and sex groups. The APC analysis showed lower relative risks in more recent periods and younger birth cohorts. The BAPC model projected substantial increases in the absolute numbers of deaths and DALYs by 2050, accompanied by increases in the corresponding age-standardized rates.

**Conclusion:**

Although age-standardized mortality and DALY rates declined from 1990 to 2023, the BAPC model projected increases in both the absolute burden and age-standardized rates of digestive system cancers attributable to the aggregate dietary risk category through 2050. These findings represent conditional model-based projections and support continued attention to sex-specific dietary-risk prevention and control strategies.

## 1 Introduction

The global and Chinese burdens from cancer have become major problems, especially those early-onset cancers (diagnosed before 50-year-olds) that attract growing interest among public health and epidemiology researchers. The burden of early-onset digestive system cancers is crucial because it is tightly related to dietary patterns, metabolic abnormalities, and socioeconomic transition (1, 2), which is when people reach their prime of education, employment, and family responsibilities. Therefore, the occurrence of cancer brings about not just premature death and disability, but also great psychological and economic stress upon families and society (3, 4). Systematically assessing the burden of early-onset digestive system cancers attributed to dietary risk is paramount for targeting disease prevention, optimal allocation of health resources, and advancing national health strategy.

Previous studies have examined the burden and temporal patterns of early-onset cancers and digestive system malignancies at the global and national levels. Several GBD-based studies have evaluated early-onset gastrointestinal cancers across different cancer sites, sexes, age groups, and geographical regions (5–8). In addition, recent studies have specifically characterized the burden and future trends of early-onset gastrointestinal cancers in the Chinese population using GBD data (9–11). The GBD 2021 Causes of Death analysis also provides the broader epidemiological framework for interpreting cause-specific mortality patterns (12). These studies have substantially advanced our understanding of early-onset gastrointestinal cancers. However, the burden of digestive system cancers specifically attributable to dietary risks has not been comprehensively evaluated across multiple cancer sites in Chinese individuals aged 25–49 years. In particular, the combined patterns of deaths and DALYs, age-standardized rates, age- and sex-specific differences, age-period-cohort effects, and long-term projections have not been examined within a single analytical framework (13).

To address this issue, we used data from the GBD study to assess the burden of digestive system cancers attributable to dietary risks among Chinese individuals aged 25–49 years. We included six cancer sites, colorectal, esophageal, stomach, liver, pancreatic, and gallbladder and biliary tract cancers, and evaluated deaths and DALYs using both absolute numbers and age-standardized rates. We used Joinpoint regression to characterize long-term temporal trends, an age-period-cohort model to estimate age, period, and cohort effects, and a Bayesian age-period-cohort model to project the disease burden through 2050. By integrating dietary-risk attribution, multiple digestive cancer sites, detailed age- and sex-specific analyses, and complementary temporal and projection methods, this study extends previous GBD-based research and provides additional evidence for targeted prevention and control strategies in China.

## 2 Methods

### 2.1 Data source and study population

Although early-onset cancer is commonly defined as cancer diagnosed before the age of 50 years, the GBD dataset used in the present study did not provide the corresponding dietary-risk-attributable estimates for individuals younger than 25 years (14). Therefore, individuals aged 0–24 years could not be included because of data availability and age-group structure limitations. We directly extracted GBD estimates of deaths and disability-adjusted life years (DALYs) attributable to the aggregate dietary risk category for colorectal, esophageal, stomach, liver, pancreatic, and gallbladder and biliary tract cancers from 1990 to 2023. The dietary risk–cancer associations were those defined in the GBD comparative risk assessment framework; no individual dietary components were separately analyzed, and no additional attributable-risk calculations were performed. Data were stratified by calendar year, sex, and five-year age groups: 25–29, 30–34, 35–39, 40–44, and 45–49 years. Because the analysis was restricted to ages 25–49 years, age-standardized mortality and DALY rates were calculated using the corresponding weights from the GBD standard population for these five age groups, with the weights normalized within the analyzed age range (15).

### 2.2 Burden description and trend analysis

We calculated the absolute numbers and age-standardized rates (ASRs) of deaths and DALYs attributable to dietary risks in 2023, together with their corresponding 95% uncertainty intervals (UIs). Temporal trends from 1990 to 2023 were analyzed using Joinpoint regression, implemented in the Joinpoint Regression Program (version 5.0.2; Statistical Research and Applications Branch, National Cancer Institute, Bethesda, MD, USA). The analysis fits a series of connected linear segments on a logarithmic scale to annual mortality and DALY rates and identifies calendar years at which the temporal trend changes significantly.

The natural logarithm of each mortality or DALY rate was modeled as the dependent variable, with calendar year as the independent variable. Separate models were fitted for the overall population, males, females, and each five-year age group. Because the study period included 34 annual observations from 1990 to 2023, the maximum number of joinpoints was set to five. The optimal number and locations of joinpoints were determined using the permutation test procedure, with an overall significance level of 0.05.

For each identified trend segment, the annual percent change (APC) was estimated from the slope of the log-linear regression model and expressed as a percentage change in the annual mortality or DALY rate. The average annual percent change (AAPC) over the entire study period was calculated as a duration-weighted average of the APC estimates for all trend segments, with the weights determined by the lengths of the corresponding segments. An APC or AAPC was considered statistically significant if its 95% confidence interval (CI) did not include zero. These analyses were used to characterize long-term trends and identify significant changes in mortality and DALY rates across sex and age subgroups (16, 17).

### 2.3 Age-period-cohort analysis

An age-period-cohort (APC) analysis was performed to estimate the independent effects of age, calendar period, and birth cohort on mortality and DALY rates. The analysis used a reference-cell parameterization to address the identification problem arising from the exact linear relationship among age, period, and birth cohort. Age group, calendar period, and birth cohort were entered into the regression model as categorical variables, with one reference category specified for each dimension. The reference categories were the 35–39-year age group, the 2004–2008 calendar period, and the 1964–1973 birth cohort. The age, period, and cohort effects were therefore interpreted relative to these reference categories. Mortality and DALY outcomes were modeled separately using a generalized linear modeling framework implemented in R software. The logarithm of the population at risk was included as an offset where applicable. The model estimated rate ratios (RRs) and corresponding 95% confidence intervals for each age group, calendar period, and birth cohort relative to its respective reference category. Because age, period, and cohort are linearly related, the reference-cell approach estimates the identifiable relative effects while avoiding the inclusion of a redundant intercept or fully unconstrained parameters. The results were used to describe differences in risk associated with age, historical period, and birth cohort, rather than to interpret the three effects as completely independent causal effects (18–20).

### 2.4 Bayesian age-period-cohort prediction

A Bayesian age-period-cohort (BAPC) model was used to project the future burden of dietary-risk-attributable digestive system cancers from 2024 to 2050. Historical age-specific mortality and DALY data from 1990 to 2023 were used to estimate future age-specific mortality and DALY rates. The model was implemented in R using the Integrated Nested Laplace Approximation (INLA) framework.

The BAPC model incorporated age, period, and cohort information to characterize temporal patterns in the age-specific mortality and DALY rates. Projections were generated separately for the overall population aged 25–49 years, males, and females. Posterior medians were used as point estimates, and the 2.5th and 97.5th percentiles of the posterior distributions were used to construct 95% uncertainty intervals. Population projections for 2024–2050 were obtained from the GBD study. The model first projected age-specific mortality and DALY rates. These projected age-specific rates were then combined with the corresponding age-specific population projections from the GBD study to calculate the annual absolute numbers of deaths and DALYs. The projected absolute numbers and age-standardized rates were subsequently summarized for the overall population aged 25–49 years and separately by sex.

The projections represent a conditional model-based scenario under the assumption that the temporal patterns observed during 1990–2023 and the GBD population projections continue through 2050. No formal internal validation, hold-out validation, or back-testing was performed to evaluate the predictive performance of the BAPC model. Therefore, the projected values should be interpreted as conditional model-based projections rather than independently validated forecasts (21–23).

## 3 Results

### 3.1 The burden in 2023

Early-onset digestive system cancers due to dietary risks caused significant burden in China in 2023. These diseases contributed to 7,134 deaths (95% UI: 2,625–12,027) and 349,764 DALYs (95% UI: 129,318–586,108) from the overall early-onset population between ages 25 and 49 years. Age-standardized mortality and DALY rates are 1.31 and 64.79 per 100,000 population, respectively. There is an obvious sex difference, where males have a much heavier burden than females. Males had 5,001 deaths (ASR: 1.78 per 100,000), which was over twice as many as female deaths at 2,133 (ASR: 0.81 per 100,000). Similarly, DALYs for males reached 245,088 (ASR: 88.13 per 100,000), while those for females were only 104,676 (ASR: 40.12 per 100,000). For each age subgroup analysis, there is a clear upward trend in the absolute numbers and rates of deaths and DALYs with age increase. The lowest burden was observed among young people (age 25–29 years), with 241 deaths (rate: 0.31 per 100,000) and 15,377 DALYs (rate: 19.72 per 100,000). The peak burden occurs in the oldest subgroup (age 45–49 years), accounting for almost half of the total early-onset burden. Deaths in this group increased sharply to 3,343 with a rate of 3.35 per 100,000, and DALYs reached 147,533 with a rate of 147.91 per 100,000. Burden remains consistently higher in males than females in every age subgroup, emphasizing the disproportionately high impact of dietary risk on early-onset digestive system cancer among the male population (Table 1 and Figure 1).

**Table 1:** The burden and trends of early-onset digestive system cancers attributable to dietary risks in China, 1990–2023.

Age	Sex	Deaths			DALYs		
		Number (95% UI)	Rate (95% UI, per 100,000)	AAPC of Rate (95% CI)	Number (95% UI)	Rate (95% UI, per 100,000)	AAPC of Rate (95% CI)
All ages (25to 49)	Both	7,134 (2,625–12,027)	1.31 (0.48–2.2)	−2.59 (−2.69 to −2.51)	3,49,764 (1,29,318–5,86,108)	64.79 (24.13–108.4)	−2.59 (−2.68 to −2.51)
	Female	2,133 (977–3,598)	0.81 (0.37–1.36)	−3.61 (−3.67 to −3.54)	1,04,676 (47,893–1,75,517)	40.12 (18.34–67.22)	−3.6 (−3.67 to −3.54)
	Male	5,001 (1,573–8,798)	1.78 (0.56–3.13)	−2.07 (−2.19 to −1.98)	2,45,088 (77,360–4,26,299)	88.13 (28.04–153.09)	−2.04 (−2.12 to −1.95)
25 to 29	Both	241 (100–403)	0.31 (0.13–0.52)	−3.48 (−3.62 to −3.38)	15,377 (6,356–25,618)	19.72 (8.15–32.85)	−3.47 (−3.6 to −3.37)
	Female	80 (33–135)	0.22 (0.09–0.37)	−4.53 (−4.66 to −4.42)	5,080 (2,118–8,565)	13.94 (5.81–23.51)	−4.53 (−4.66 to −4.41)
	Male	161 (57–277)	0.39 (0.14–0.67)	−2.79 (−2.92 to −2.68)	10,298 (3,644–17,596)	24.78 (8.77–42.34)	−2.78 (−2.91 to −2.66)
30 to 34	Both	655 (254–1,053)	0.61 (0.24–0.98)	−2.94 (−3.07 to −2.81)	38,535 (14,983–61,856)	35.87 (13.95–57.58)	−2.92 (−3.04 to −2.78)
	Female	205 (98–322)	0.4 (0.19–0.63)	−4.05 (−4.16 to −3.95)	12,004 (5,747–18,854)	23.54 (11.27–36.97)	−4.04 (−4.16 to −3.94)
	Male	451 (149–768)	0.8 (0.26–1.36)	−2.21 (−2.36 to −2.04)	26,531 (8,796–45,228)	47.02 (15.59–80.16)	−2.18 (−2.33 to −2.02)
35 to 39	Both	1,241 (471–2,054)	1.05 (0.4–1.74)	−2.52 (−2.64 to −2.4)	67,160 (25,479–1,10,930)	56.81 (21.55–93.83)	−2.49 (−2.61 to −2.37)
	Female	376 (161–630)	0.65 (0.28–1.1)	−3.6 (−3.7 to −3.51)	20,271 (8,709–33,871)	35.31 (15.17–59)	−3.57 (−3.68 to −3.47)
	Male	865 (259–1,471)	1.42 (0.43–2.42)	−1.89 (−2 to −1.76)	46,889 (14,057–79,400)	77.11 (23.12–130.57)	−1.85 (−1.96 to −1.72)
40 to 44	Both	1,654 (645–2,754)	1.82 (0.71–3.03)	−2.62 (−2.7 to −2.53)	81,159 (31,646–1,34,964)	89.24 (34.8–148.4)	−2.58 (−2.67 to −2.49)
	Female	494 (233–841)	1.12 (0.53–1.9)	−3.55 (−3.65 to −3.46)	24,186 (11,419–41,195)	54.68 (25.82–93.14)	−3.53 (−3.63 to −3.43)
	Male	1,160 (401–2,006)	2.48 (0.86–4.29)	−2 (−2.1 to −1.9)	56,973 (19,711–98,434)	121.95 (42.19–210.7)	−1.96 (−2.07 to −1.85)
45 to 49	Both	3,343 (1,155–5,763)	3.35 (1.16–5.78)	−2.57 (−2.71 to −2.45)	1,47,533 (50,854–2,52,740)	147.91 (50.98–253.39)	−2.53 (−2.66 to −2.43)
	Female	979 (451–1,669)	2 (0.92–3.4)	−3.43 (−3.57 to −3.29)	43,136 (19,901–73,032)	87.94 (40.57–148.89)	−3.38 (−3.52 to −3.23)
	Male	2,364 (706–4,276)	4.66 (1.39–8.43)	−1.99 (−2.19 to −1.84)	1,04,397 (31,152–1,85,641)	205.94 (61.45–366.2)	−1.95 (−2.13 to −1.8)

**Figure 1:**
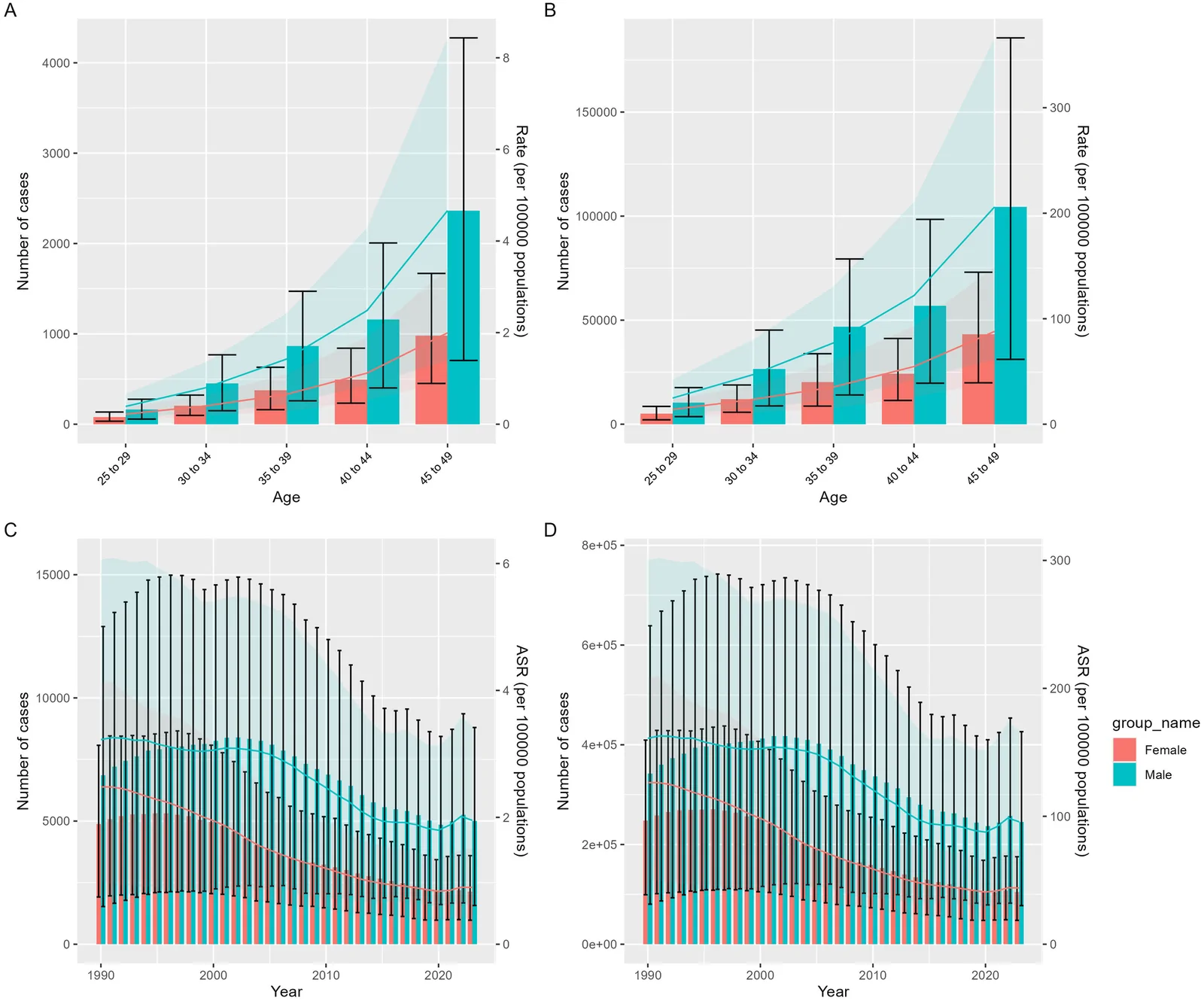
Disease burden of early-onset digestive system cancers attributable to dietary risks in China by age group and year, 1990–2023. The bar charts display the absolute number of cases (left *y*-axis) stratified by sex (Salmon: female; teal: male), and the overlaid line graphs depict the corresponding age-standardized rates (ASR, right *y*-axis) with 95% uncertainty intervals (shaded areas). **(A)** Deaths by five-year age group (25–49 years) in 2023. **(B)** DALYs by five-year age group (25–49 years) in 2023. **(C)** Temporal trends in the number of deaths and ASR of mortality from 1990 to 2023. **(D)** Temporal trends in the number of DALYs and ASR of DALY rate from 1990 to 2023. Error bars represent 95% uncertainty intervals. ASR, age-standardized rate; DALYs, disability-adjusted life years.

### 3.2 Trends from 1990 to 2023

From 1990 to 2023, the ASRs of deaths and DALYs attributable to dietary risks among the early-onset population (aged 25–49 years) in China demonstrated a sustained and statistically significant declining trend. For the overall population, the AAPC of the mortality rate was −2.59% (95% CI: −2.69% to −2.51%), and that of the DALY rate was similarly −2.59% (95% CI: −2.68% to −2.51%), indicating a consistent and substantial reduction in the burden over the study period.

A notable sex difference was observed in the pace of decline. Females experienced a more rapid decrease in both mortality and DALY rates, with AAPCs of −3.61% (95% CI: −3.67% to −3.54%) and −3.60% (95% CI: −3.67% to −3.54%), respectively. In contrast, the corresponding AAPCs in males were more moderate, at −2.07% (95% CI: −2.19% to −1.98%) for mortality and −2.04% (95% CI: −2.12% to −1.95%) for DALYs, suggesting that while the overall burden declined in both sexes, the reduction was considerably slower among males.

Across age subgroups, all five five-year age groups exhibited statistically significant downward trends in both mortality and DALY rates throughout the study period. The most pronounced decline was observed in the youngest age group (25–29 years), with an AAPC of −3.48% for mortality and −3.47% for DALYs. The rate of decline was somewhat attenuated in the middle age groups, with the 35–39 age group recording the slightest steep decrease among all subgroups (AAPC: −2.52% for mortality and −2.49% for DALYs). The 40–44 and 45–49 age groups showed comparable declining trends, with AAPCs of approximately −2.60% and −2.57% for mortality, respectively. Taken together, these findings indicate that the burden of early-onset digestive system cancers attributable to dietary risks has declined broadly across all age and sex strata in China over the past three decades, although the magnitude of improvement varied by subgroup (Table 1, Figures 1, 2).

**Figure 2:**
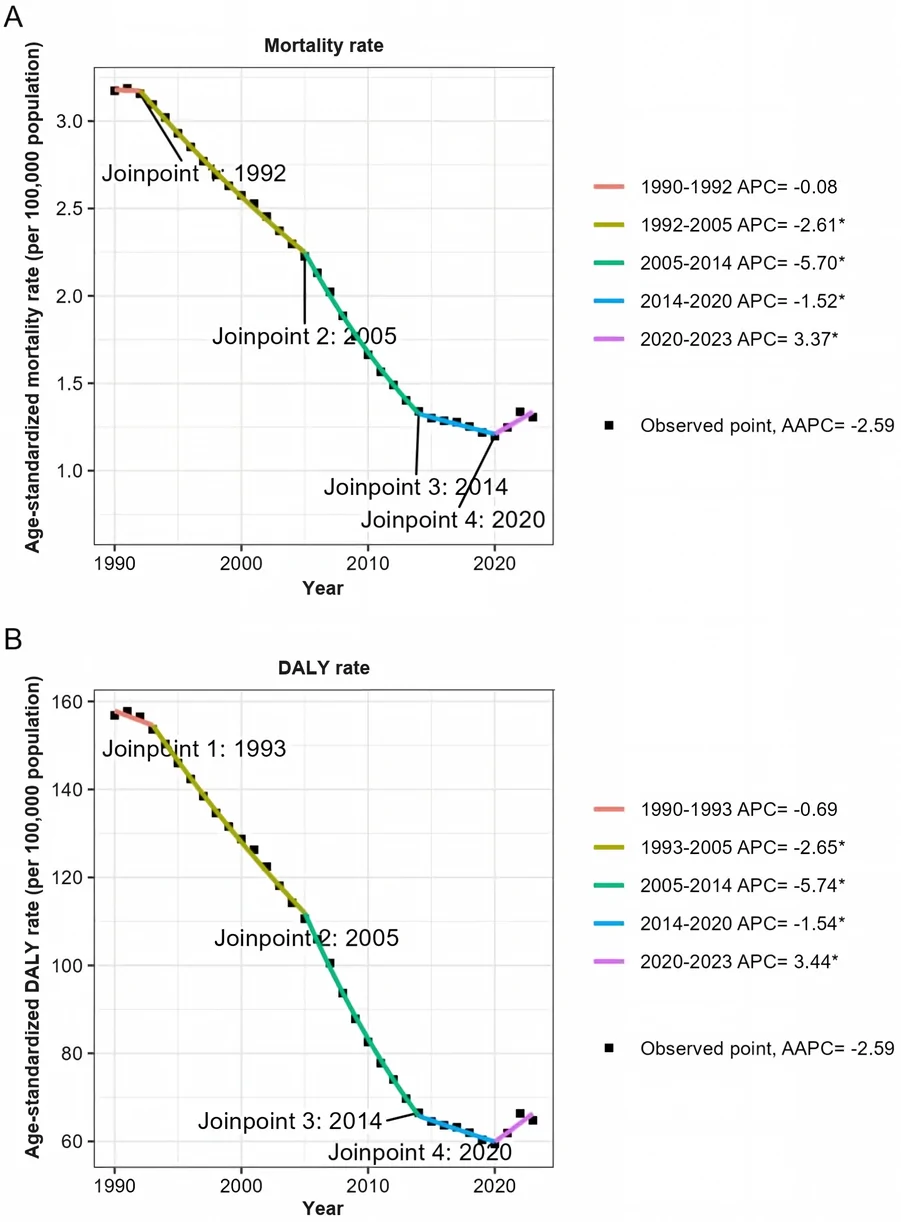
Joinpoint regression analysis of age-standardized rates of early-onset digestive system cancers attributable to dietary risks in China, 1990–2023. Colored line segments represent distinct trend intervals identified by the Joinpoint regression model, with each segment annotated with the corresponding annual percent change (APC). Black squares indicate observed data points. The overall average annual percent change (AAPC) for the entire study period is provided in the legend. Asterisks (*) denote statistically significant APCs (*P* < 0.05). **(A)** Age-standardized mortality rate. **(B)** Age-standardized DALY rate. APC, annual percent change; AAPC, average annual percent change; DALYs, disability-adjusted life years.

### 3.3 Age-period-cohort model

Age effect on the burden of early-onset digestive system cancers due to dietary risks showed an increasing trend with aging. Taking the 35 to 39 age group as reference (RR = 1.00), the risk of death or DALY is much less among young age groups. For example, the mortality RR was 0.54 (95% CI: 0.51–0.57) and the DALY RR was 0.64 (95% CI: 0.61–0.67) in the 25 to 29 age group. On the other hand, the risk sharply increases in the older subgroups, reaching peak value in the 45 to 49 age group, when the mortality RR reaches up to 2.07 (95% CI: 2.00–2.14) and the DALY RR reaches up to 1.70 (95% CI: 1.64–1.75). These results demonstrate an age-related pattern in mortality and DALY risks, with the highest relative risks observed in the 45–49-year age group (Figure 3).

**Figure 3:**
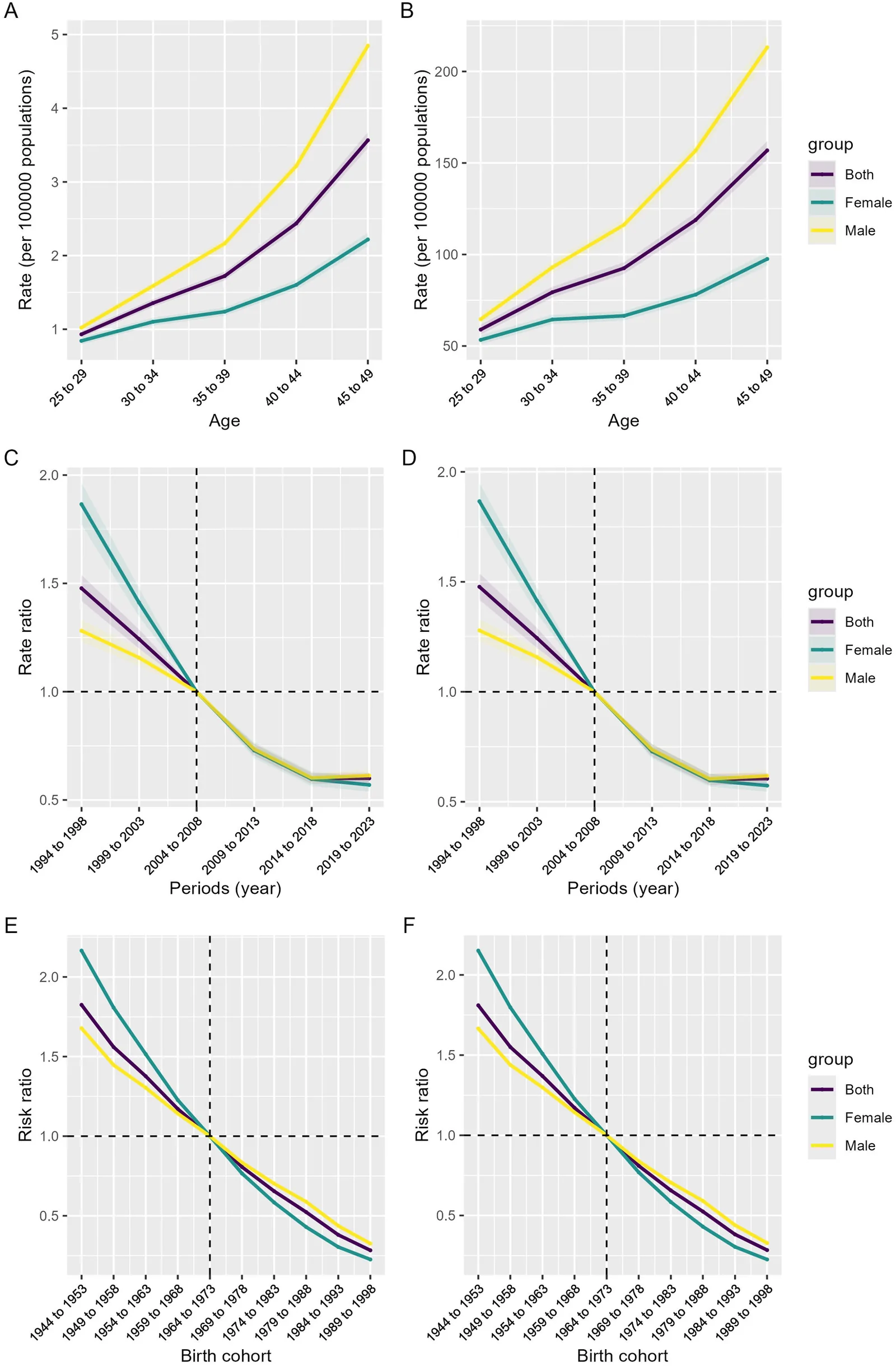
Age-period-cohort analysis of early-onset digestive system cancers attributable to dietary risks in China. Results are stratified by sex (purple: both sexes combined; teal: female; yellow: male). Shaded areas represent 95% confidence intervals. Dashed lines indicate the reference category (rate ratio = 1.00). **(A)** Age effect on mortality rate (per 100,000 population) across five-year age groups (25–49 years). **(B)** Age effect on DALY rate (per 100,000 populations) across five-year age groups (25–49 years). **(C)** Period effect expressed as rate ratios for mortality, with 2004–2008 as the reference period. **(D)** Period effect expressed as rate ratios for DALYs, with 2004–2008 as the reference period. **(E)** Cohort effect expressed as risk ratios for mortality, with the 1964–1973 birth cohort as the reference. **(F)** Cohort effect expressed as risk ratios for DALYs, with the 1964–1973 birth cohort as the reference. DALYs, disability-adjusted life years.

For the period effect, there is an evident decline across all study decades. When we used the 2004 to 2008 period as our reference point with RR = 1.00, much greater risks are found in previous periods. The mortality and DALY RRs from the 1994 to 1998 period were both 1.48. The risk ratio decreases gradually through time down to 0.73 for both mortality and DALYs during the 2009 to 2013 period. This trend continues even in the most recent period (2019 to 2023). The mortality RR declines further to 0.60 (95% CI: 0.58–0.63) while DALY RR reaches 0.61 (95% CI: 0.58–0.63). This finding indicates a decline in the modeled mortality and DALY risks across successive calendar periods, with the lowest relative risks observed in the most recent period (2019–2023) (Figure 3).

We observed a positive trend of decreasing disease burden across successive younger birth cohorts. When compared with our reference cohort born between 1964 and 1973 (RR = 1.00), older birth cohorts had significantly increased risks. RR values for the first cohort (born between 1944 and 1953) were as high as 1.82 (95% CI: 1.73–1.93) for mortality and 1.81 (95% CI: 1.71–1.92) for DALYs. Risk was gradually decreasing over time for newer birth cohorts. Mortality and DALY RRs for the youngest cohort (born between 1989 and 1998) plummeted to 0.28 (95% CI: 0.24–0.34) and 0.28 (95% CI: 0.24–0.33), respectively. These results indicate lower modeled mortality and DALY risks in more recent birth cohorts compared with the reference cohort, representing a cohort-related pattern in the data (Figure 3).

### 3.4 BAPC prediction

Based on the BAPC model, the burden of early-onset digestive system cancers attributable to dietary risks in China is projected to experience an overall increase by 2050. For the overall early-onset population, the number of deaths is predicted to rise from 7,821 (95% UI: 7,158–8,485) in 2024 to 13,223 in 2050, with the corresponding ASR increasing from 1.35 to 1.88 per 100,000 population. Similarly, the total DALYs are projected to nearly double, growing from 387,301 (95% UI: 356,498–418,103) in 2024 to 750,729 in 2050, while the DALY ASR is expected to climb from 67.67 to 105.63 per 100,000 population. A persistent sex disparity is also anticipated throughout the projection period. In males, the projected number of deaths will increase from 5,430 (ASR: 1.85) in 2024 to 10,303 (ASR: 2.34) in 2050, and DALYs will rise from 269,188 (ASR: 92.48) to 617,524 (ASR: 137.42). For females, although the absolute burden remains lower than that of males, an upward trend is still evident; the number of deaths is expected to grow from 2,297 (ASR: 0.81) in 2024 to 3,200 (ASR: 1.04) in 2050, and DALYs will increase from 115,271 (ASR: 41.14) to 187,879 (ASR: 61.94). These projections indicate that without intensified and targeted interventions, the health and socioeconomic impact of these cancers will continue to escalate over the next few decades (Figure 4).

**Figure 4:**
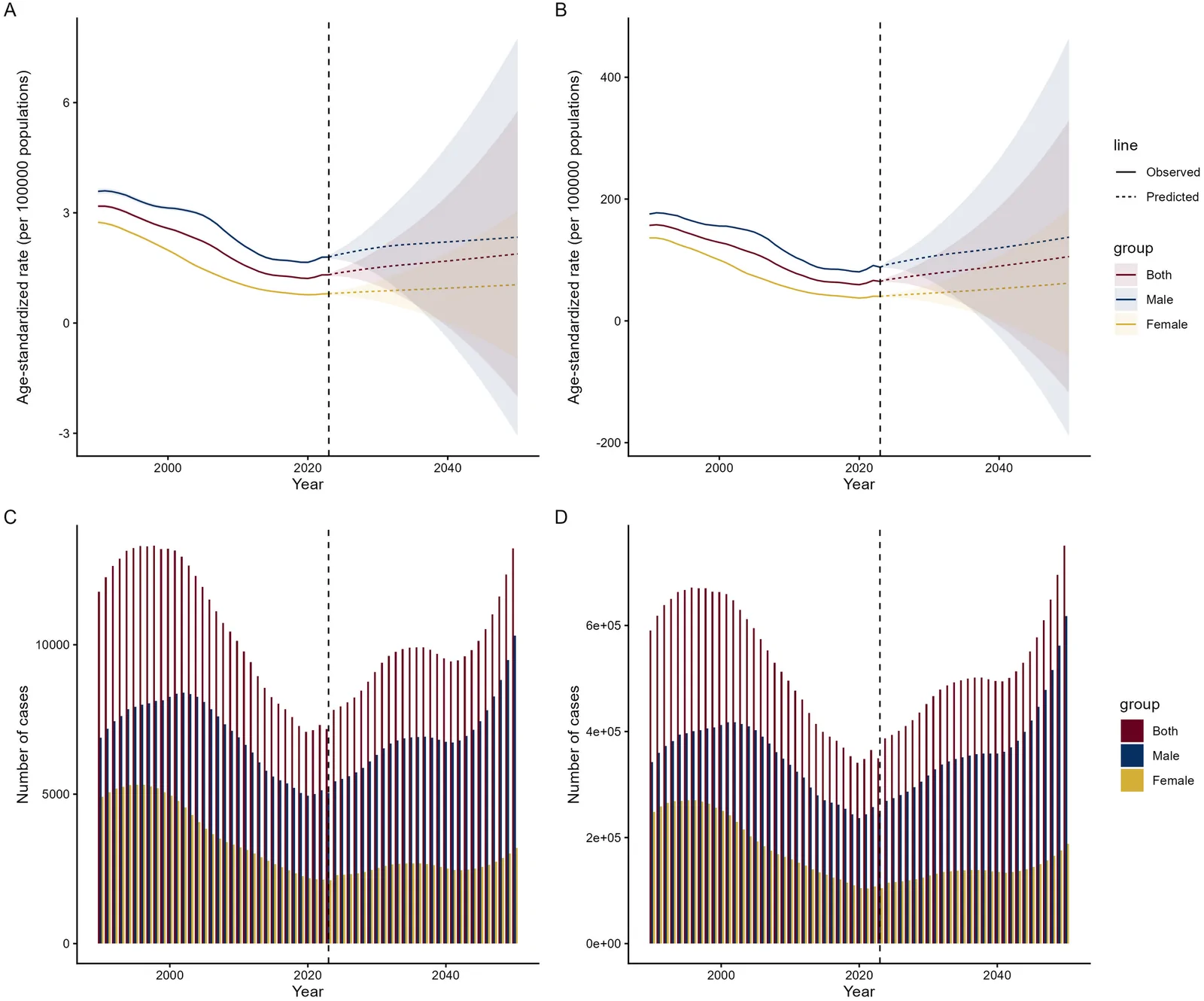
Bayesian age-period-cohort (BAPC) model projections of early-onset digestive system cancers attributable to dietary risks in China, 1990–2050. The vertical dashed line demarcates the observed period (left) from the projected period (right). Solid lines represent observed data and dashed lines represent predicted values; shaded areas indicate 95% uncertainty intervals for projections. Groups are color-coded (red/dark red: both sexes combined; blue/dark blue: male; yellow/gold: female). **(A)** Projected age-standardized mortality rate (per 100,000 populations) from 1990 to 2050. **(B)** Projected age-standardized DALY rate (per 100,000 populations) from 1990 to 2050. **(C)** Projected absolute number of deaths from 1990 to 2050, stratified by sex. **(D)** Projected absolute number of DALYs from 1990 to 2050, stratified by sex. BAPC, Bayesian Age-Period-Cohort; ASR, age-standardized rate; DALYs, disability-adjusted life years.

## 4 Discussion

We systematically assessed the burden, trends, and future projection of early-onset digestive system cancers attributed to diet risk in China between 1990 and 2023. We found that although the ASRs for mortality and DALYs have significantly decreased during the last three decades, the absolute burden is still considerable, with marked male predominance and alarmingly escalating by 2050. This highlights the double challenge of dealing with historical burden as well as the impending surge due to demographic and lifestyle transition.

The decline in age-standardized mortality and DALY rates between 1990 and 2023, together with the period and cohort patterns observed in the APC analysis, may be consistent with broad changes in socioeconomic conditions, healthcare access, screening practices, and public health policies in China (24–27). However, these factors were not directly measured or evaluated in the present study, and their specific contributions to the observed decline cannot be established from our analyses. Similarly, the lower relative risks observed in more recent birth cohorts may reflect differences in early-life conditions, dietary exposures, healthcare access, or health awareness across generations (9, 19, 20). The persistent male predominance warrants attention. Previous studies have suggested that differences in dietary habits, metabolic risk profiles, occupational exposures, and healthcare-seeking behavior may contribute to sex-related disparities in digestive cancer burden (28, 29). However, these factors were not directly evaluated in the present study, and their contributions cannot be determined from our data. Moreover, the BAPC model projected increases in both the absolute numbers of deaths and DALYs and their corresponding age-standardized rates between 2024 and 2050. Mortality ASR was projected to increase from 1.35 to 1.88 per 100,000 population, while DALY ASR was projected to increase from 67.67 to 105.63 per 100,000 population. These findings indicate that the projected increase cannot be attributed to demographic changes alone. Because no formal decomposition of demographic and rate components was performed, the projections should be interpreted as conditional model-based estimates under the assumed continuation of historical temporal patterns and projected population trends (22, 23, 30).

In comparison to recent global and regional studies, we share the general agreement on increasing influence from modifiable lifestyle factors for early-onset gastrointestinal cancers (1, 2, 5, 6, 17). However, different from studies in Western populations showing surging incidence rates of early-onset colorectal cancer (7, 8, 31–34), our APC analyses show consistently decreasing overall risk of diet-related digestive cancers over recent cohorts in China. This difference reflects the distinct epidemiological transition in China, which was traditionally controlled through traditional risk factors (undernutrition, specific infections), but at present faces threats from westernized diets and rising obesity rates (10, 11, 35–38). Innovation of our study includes integrating various digestive cancer types comprehensively (14, 15, 39–42) as well as a robust prediction model specially designed for the Chinese early-onset population.

These findings have significant academic and practical implications: by quantifying the trajectory of diet-related early-onset digestive cancers, we provide an essential evidence basis for policymakers to pivot from generalized cancer prevention toward targeted, age- and sex-specific intervention. Public health efforts need to target the male demographic with mitigation of dietary risks via community education, stringent food policies, as well as expanding early screening programs tailored for younger adults. Future work needs to dive deeper into the specific dietary components (e.g., high red meat consumption, low fiber intake), which drive these trends and assess the cost-effectiveness of targeted screening within the under-50 population.

The six cancer types included in this study have different epidemiological characteristics and may not share identical temporal patterns. Therefore, the aggregate estimates presented here should be interpreted as the overall burden of dietary-risk-attributable digestive system cancers rather than as evidence that the observed trend applies uniformly to colorectal, stomach, esophageal, liver, pancreatic, and gallbladder and biliary tract cancers. The overall pattern may be influenced disproportionately by specific cancer types with a larger absolute burden.

Several limitations should be considered. First, this study relied on modeled GBD estimates rather than directly observed individual-level data. The quality, completeness, and geographic coverage of the underlying data may have varied across years, age groups, and population subgroups, which may affect the precision of some estimates. Second, the attribution of cancer burden to dietary risks was based on the GBD comparative risk assessment framework and is subject to uncertainty in dietary exposure measurement, exposure–outcome relationships, and the assumptions used to estimate population-attributable burden. Because we analyzed the aggregate dietary risk category, the contributions of individual dietary components could not be evaluated. Third, the six cancer types included in the aggregate analysis may have different etiological and temporal patterns, and the overall trend may therefore mask cancer-specific heterogeneity. Fourth, the Joinpoint, APC, and BAPC models describe associations and projections but cannot establish causal relationships. Finally, the projections through 2050 assume continuation of historical temporal patterns and GBD population projections. Future changes in dietary patterns, obesity prevalence, screening, diagnostic approaches, treatments, healthcare access, and public health policies may cause the actual burden to differ from the projected estimates. No formal internal validation or back-testing was performed; therefore, the projections should be interpreted as conditional model-based estimates rather than definitive forecasts.

## 5 Conclusion

Although the age-standardized rates of digestive system cancers attributable to dietary risks decreased in China from 1990 to 2023, the BAPC model projects that both the absolute burden and age-standardized rates may increase through 2050, particularly among males. These findings represent model-based projections under specified assumptions and support continued attention to sex-specific, targeted dietary-risk prevention strategies.

## Data Availability

The datasets presented in this study can be found in online repositories. The names of the repository/repositories and accession number(s) can be found below: Global Burden of Disease (GBD) Study. Data available from: https://vizhub.healthdata.org/gbd-results/No accession number is applicable for this publicly available aggregated population dataset.
